# Wavefront‐Like LAM Method: A Case Report of Residual Potential Identification Using Local Voltage Derived From Late Annotation Mapping

**DOI:** 10.1002/joa3.70353

**Published:** 2026-05-02

**Authors:** Tomoyoshi Morioku, Yasuyuki Egami, Yasuharu Matsunaga‐Lee, Masamichi Yano, Masami Nishino

**Affiliations:** ^1^ Clinical Engineering Technologist, Osaka Rosai Hospital Osaka Japan; ^2^ Division of Cardiology Osaka Rosai Hospital Osaka Japan

**Keywords:** atrial fibrillation, late annotation mapping, local voltage, posterior wall isolation, voltage mapping

## Abstract

The Wavefront‐like LAM method preserves wavefront‐based timing consistency while enabling quantitative local voltage evaluation. This method improves differentiation between low‐voltage and no‐voltage regions and suggests that residual potentials appearing isolated on conventional voltage mapping can be identified.

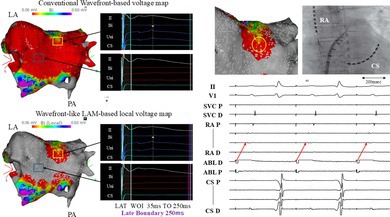

Voltage mapping is essential for characterizing the atrial substrate; however, conventional wavefront‐based voltage maps (WVM) display signals below the lower threshold in red, blurring distinctions between no‐voltage (true scar) and low‐voltage regions and increasing susceptibility to far‐field potentials (FP) and environmental noise, potentially causing misclassification or suppression [1]. The CARTO system (Biosense Webster, Diamond Bar, CA, USA) incorporates a Late Annotation Mapping (LAM) module that calculates “Local Voltage”. Originally designed for late potential (LP) detection in ventricular mapping, LAM performs annotations by backward searching from the end of a predefined window of interest (WOI) toward the Late Boundary. In voltage mapping, LAM extracts electrograms before and after the annotation point and calculates their maximum amplitude as local voltage, thereby attenuating the influence of FP and noise. However, because LAM‐based activation maps are optimized for LP detection, their annotation criteria are less strict than conventional wavefront annotation, which may reduce the temporal consistency of local activation times (LAT). To address this limitation and focus on accurate voltage evaluation, we developed the “Wavefront‐like LAM method,” which preserves wavefront‐based timing consistency while utilizing LAM‐derived local voltage. Using this method, residual potentials within the posterior wall isolation (PWI) area were successfully identified in this case, and targeted ablation at these sites achieved complete isolation.

An 83‐year‐old woman with persistent atrial fibrillation (AF) and heart failure underwent catheter ablation. Three‐dimensional mapping was performed using the CARTO system, followed by pulmonary vein isolation (PVI) and PWI. A BeeAT catheter (Japan Lifeline) was positioned in the coronary sinus, an OCTARAY catheter (Biosense Webster) was used for mapping, and a QDOT MICRO catheter (Biosense Webster) was used for ablation. After PVI and PWI, voltage mapping was performed with the OCTARAY catheter. Using the CARTO Replay function, a conventional wavefront‐based voltage map (WVM) and a Wavefront‐like LAM‐based local voltage map (LVM) were generated and compared, with thresholds of 0.05–0.5 mV.

For the LAM configuration, the WOI was set from 35 ms to 250 ms, and the Wavefront‐like LAM method was defined by aligning the Late Boundary with the end of the WOI (250 ms) and applied in this case. On WVM, the entire PWI region was uniformly displayed in red, whereas LVM showed red areas localized mainly along the roof line and discrete yellow sites suggestive of residual potentials (Figure 1A).

**FIGURE 1:**
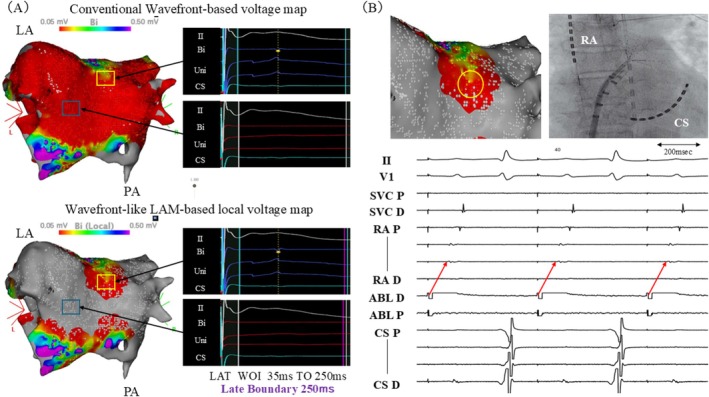
(A) Comparison between the conventional Wavefront‐based voltage map (upper panel) and the Wavefront‐like LAM‐based local voltage map (lower panel). The voltage map settings were 0.5 mV (upper limit) and 0.05 mV (lower limit). The conventional Wavefront‐based voltage map displayed the entire posterior wall isolation region uniformly in red, whereas the LAM‐based local voltage map showed red areas localized near the roof line, suggesting the presence of residual potentials (yellow squares). Representative electrograms are shown for the annotated sites in both maps at the location indicated by the blue box, where no local electrogram was observed. These sites appear red on the WVM but gray on the LVM. (B) Pacing was performed at the site where residual potentials were suggested (yellow circle) using the ablation catheter at 1 ms/20 V. The corresponding catheter position and electrogram are shown. ABL, Ablation catheter; Bi, Bipolar; CS, Coronary sinus; D, Distal; LA, Left atrium; LAM, Late Annotation Mapping; LAT, Local activation time; P, Proximal; PA, Posterior–anterior view; RA, Right atrium; SVC, Superior vena cava; Uni, Unipolar; WOI, Window of interest.

At the site indicated by the blue box on both maps, no meaningful local electrograms were observed. The signals were extremely low‐amplitude and below the level of identical local electrograms. However, this area appeared red on the WVM but gray on the LVM. Among 110 points, LVM identified 5 yellow (4%), 54 red (49%), and 51 gray points (46%), reclassifying many red areas on WVM as gray or yellow. High‐output pacing (1 ms/20 V) at a yellow site captured the left atrium, confirming residual potentials (Figure 1B). Additional ablation at this site converted the local voltage to gray (Figure 2A), and repeat pacing demonstrated exit block (Figure 2B), confirming complete PWI isolation. Conventional WVM is affected by FPs and environmental noise and cannot distinguish low‐voltage from no‐voltage areas because signals below the threshold are displayed in red, potentially overestimating substrate. In contrast, the LVM calculates local voltage based on electrogram amplitudes immediately before and after the annotation point. This algorithm is less susceptible to FP interference and allows clear differentiation between low‐voltage and no‐voltage regions. Nearly half of the regions labeled red on the WVM were reclassified as no‐voltage (gray) by LVM, whereas the remaining red and yellow points indicated preserved low‐amplitude local potentials. Notably, yellow points identified viable local electrograms that were misclassified as low voltage on the WVM. These findings suggest that the LVM algorithm provides a more accurate reflection of true local voltage than WVM and improves substrate evaluation. Although activation maps may visually resemble the LVM in that no‐LAT areas appear gray, the underlying meaning of these gray areas differs fundamentally between the two mapping approaches. In activation maps, gray regions represent sites where local activation time cannot be assigned, which may occur not only in true no‐voltage areas but also in regions with very low‐amplitude signals that fail to meet annotation criteria. Therefore, gray areas on activation maps do not necessarily indicate the absence of viable myocardial tissue and reflect a binary representation of signal annotation (presence or absence of activation).

**FIGURE 2:**
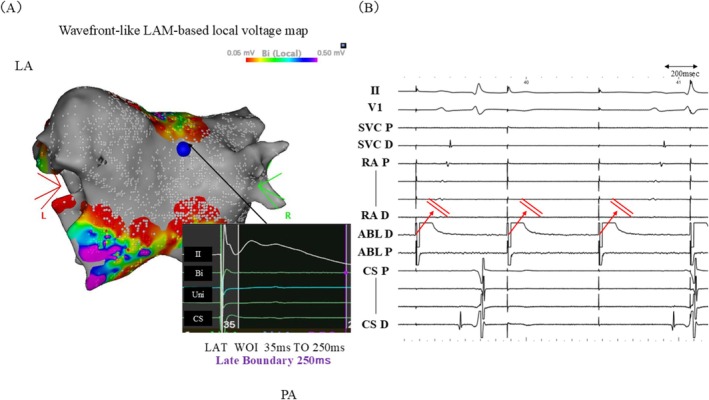
(A) The Wavefront‐like LAM‐based local voltage map reconstructed after additional ablation at the site where residual potentials were confirmed (blue circle). The site changed to gray, indicating disappearance of local voltage. (B) Pacing from the same site at 1 ms/20 V confirmed the presence of an exit block. No conduction to the left atrium was observed. ABL, Ablation catheter; Bi, Bipolar; CS, Coronary sinus; D, Distal; LA, Left atrium; LAM, Late Annotation Mapping; LAT, Local activation time; P, Proximal; PA, Posterior–anterior view; RA, Right atrium; SVC, Superior vena cava; Uni, Unipolar; WOI, Window of interest.

In contrast, in the LVM, gray regions indicate the absence of detectable local voltage within the defined window of interest. Because the LVM allows quantitative assessment of electrogram amplitude under consistent annotation conditions, it enables differentiation between true no‐voltage areas and very low‐voltage regions. This distinction may be clinically important for identifying residual viable tissue within regions that appear electrically silent on conventional mapping. Although lowering the conventional threshold (e.g., to 0.01 mV) may theoretically allow detection of subtle signals, it increases susceptibility to noise. In the present case, this adjustment did not result in a meaningful difference, whereas LVM provided clearer visualization of residual potentials (Figure S1).

In this case, a localized color discrepancy was observed near the bottom line of the PWI region on the LAM‐based activation map (Figure 3A, red circles), which was not present on the wavefront‐based activation map (Figure 3B). In the conventional LAM‐based activation map, the late annotation algorithm is applied from the end of the WOI to the Late Boundary, and the wavefront algorithm from the Late Boundary to the beginning of the WOI (Figure 3C).

**FIGURE 3:**
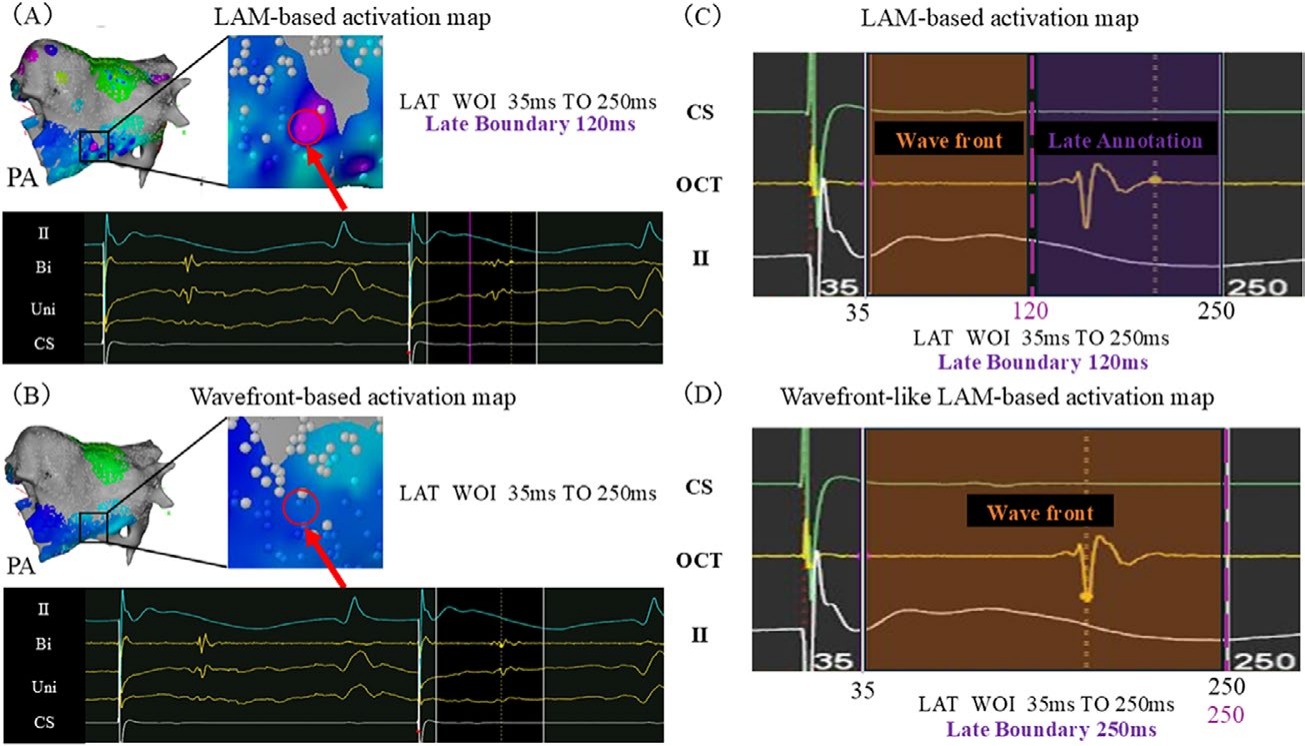
(A) LAM‐based activation map during PWI. A localized color discrepancy was observed near the bottom line region (red circles) compared with the Wavefront‐based activation map in (B) (upper panel). Red arrows indicate the corresponding local electrogram annotations (lower panel). (B) Wavefront‐based activation map during PWI (upper panel). Red arrows indicate the corresponding local electrogram annotations (lower panel). (C) LAM‐based activation map. The settings of the LAM‐based activation map used in this case (A). The WOI was set from 35 ms to 250 ms, and the Late Boundary was set at 120 ms. The Late Annotation algorithm was applied from the end of the WOI to the Late Boundary, and the Wavefront algorithm from the Late Boundary to the beginning of the WOI. At this point, because the Late Annotation algorithm was applied, the electrogram was annotated at the later phase of the recorded potential. (D) Wavefront‐like LAM‐based activation map. Configuration of the Wavefront‐like LAM method. The WOI was set from 35 ms to 250 ms, with the Late Boundary aligned with the end of the WOI (250 ms). This setting maintained identical conditions to the Wavefront‐based activation map across the entire WOI, enabling consistent activation mapping while allowing evaluation of LAM‐based local voltage. At this point, because the wavefront‐based activation map was applied, the electrogram was annotated at the timing of the maximal negative dV/dt on the unipolar electrogram of the recorded potential. Bi, Bipolar; CS, Coronary sinus; II, Surface ECG lead II; LAM, Late Annotation Mapping; LAT, Local activation time; OCT, OCTARAY catheter; PA, Posterior–anterior view; PWI, Posterior wall isolation; Uni, Unipolar; WOI, Window of interest.

In contrast, the Wavefront‐like LAM method was designed to preserve the consistency of the wavefront‐based activation map and maintain a clear distinction between low‐ and no‐voltage areas. By aligning the Late Boundary with the end of the WOI, uniform wavefront‐based criteria are applied across the WOI, preserving timing consistency while enabling local voltage computation (Figure 3D).

In this case, residual potentials were identified by LVM in an area that appeared completely isolated on WVM during PWI, and additional ablation achieved complete isolation. Thus, LVM—especially with the Wavefront‐like LAM method—visualizes residual potential while maintaining activation map consistency.

## Funding

The authors have nothing to report.

## Ethics Statement

The authors have nothing to report.

## Consent

The patient provided written informed consent for the ablation procedures and agreed to the publication of his case details and images in this report.

## Conflicts of Interest

The authors declare no conflicts of interest.

## Supporting information


**Figure S1:** Comparison of conventional voltage maps with different lower voltage thresholds. The lower threshold was set at 0.05 mV (left panel) and 0.01 mV (right panel) with the same upper threshold (0.50 mV). The black box indicates the region of interest. In this area, the distribution of voltage points showed only minor differences between the two settings. In the black box region, among a total of 56 points, 45 red (80%), 8 orange (14%), and 3 yellow (5%) points were identified with the 0.05 mV threshold, whereas 35 red (62%), 10 orange (17%), 7 yellow (12%), and 1 green (1%) points were identified with the 0.01 mV threshold. Overall, no substantial difference in the voltage map was observed between the two threshold settings.

## Data Availability

The data that support the findings of this study are available on request from the corresponding author. The data are not publicly available due to privacy or ethical restrictions.
