# Supplementary figures and images for: Wavefront‐Like LAM Method: A Case Report of Residual Potential Identification Using Local Voltage Derived From Late Annotation Mapping
Source: J Arrhythm. 2026 May 2;42(3):e70353. doi: 10.1002/joa3.70353 (PMC13135172; doi:10.1002/joa3.70353)

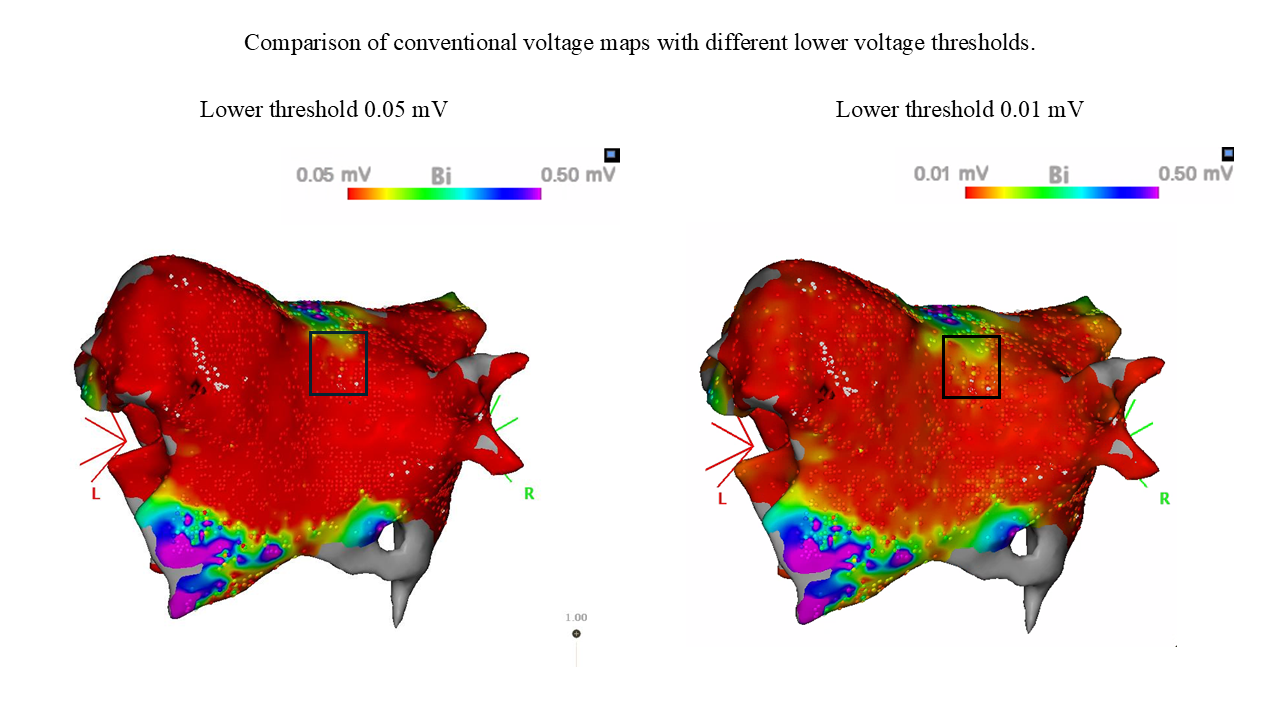

Supplement: Supplementary file 1 — Figure S1: Comparison of conventional voltage maps with different lower voltage thresholds. The lower threshold was set at 0.05 mV (left panel) and 0.01 mV (right panel) with the same upper threshold (0.50 mV). The black box indicates the region of interest. In this area, the distribution of voltage points showed only minor differences between the two settings. In the black box region, among a total of 56 points, 45 red (80%), 8 orange (14%), and 3 yellow (5%) points were identified with the 0.05 mV threshold, whereas 35 red (62%), 10 orange (17%), 7 yellow (12%), and 1 green (1%) points were identified with the 0.01 mV threshold. Overall, no substantial difference in the voltage map was observed between the two threshold settings. [file JOA3-42-e70353-s001.tif]
